# Correlates of weight status among Norwegian 11-year-olds: The HEIA study

**DOI:** 10.1186/1471-2458-12-1053

**Published:** 2012-12-06

**Authors:** May Grydeland, Ingunn H Bergh, Mona Bjelland, Nanna Lien, Lene F Andersen, Yngvar Ommundsen, Knut-Inge Klepp, Sigmund A Anderssen

**Affiliations:** 1 Department of Nutrition, Faculty of Medicine, University of Oslo, Oslo, Norway; 2 Department of Sports Medicine, Norwegian School of Sport Sciences, PB 40 Ullevaal Stadion, NO-0807, Oslo, Norway; 3 Department of Coaching and Psychology, Norwegian School of Sport Sciences, Oslo, Norway

**Keywords:** Overweight, Physical activity, Sedentary time, Parental education, Diet, Children, Adolescents

## Abstract

**Background:**

The underlying mechanisms of overweight and obesity in adolescents are still not fully understood. The aim of this study was to investigate modifiable and non-modifiable correlates of weight status among 1103 Norwegian 11-year-old adolescents in the HEalth in Adolescents (HEIA) study, including demographic factors such as gender and parental education, and behavioral factors such as intake of sugar-sweetened beverages, snacks and breakfast consumption, watching TV and playing computer games, physical activity and sedentary time.

**Methods:**

Weight and height were measured objectively, body mass index (BMI) was calculated and International Obesity Task Force cut-offs were used to define weight status. Physical activity and sedentary time were measured by accelerometers. Other behavioral correlates and pubertal status were self-reported by questionnaires. Parental education was reported by the parents on the consent form for their child. Associations were investigated using logistic regressions.

**Results:**

There were gender differences in behavioral correlates of weight status but not for weight status itself. Adolescents with parents in the highest education category had a 46% reduced odds of being overweight compared to adolescents with parents in the lowest education category. Adolescents with parents with medium education had 42% lower odds of being overweight than adolescents with parents with the lowest education category. Level of parental education, breakfast consumption and moderate to vigorous physical activity were positively associated with being normal weight, and time watching TV was positively associated with being overweight for the total sample. Gender differences were detected; boys had a doubled risk of being overweight for every additional hour of watching TV per week, while for girls there was no association.

**Conclusions:**

The present study showed a social gradient in weight status in 11-year-olds. Both breakfast consumption and moderate to vigorous physical activity were inversely associated with weight status. No associations were found between intake of sugar-sweetened beverages and snacks, playing computer games and weight status. Watching TV was positively associated with weight status for boys but not for girls. Interventions are needed to gain more insight into the correlates of change in weight status.

## Background

Overweight and obesity in children and adolescents have been associated with several health risks and social consequences [1], and often seem to follow into adulthood [1-3]. Over the last decades the proportions of overweight and obesity among children and adolescents have increased both in developed countries, including Norway, and in several developing countries [4-6]. Recently, however, a leveling-off has been observed in this development in some European countries, but this trend seems not to have reached the lower socio-economic groups equally well as the more benefited groups of society [7,8].

Identifying modifiable correlates of overweight/obesity in specific age-groups is important to be able to develop intervention strategies targeting the most important weight determinants in order to combat the overweight epidemic. Overweight and obesity in children and adolescents have been associated with certain dietary behaviors, physical activity and sedentary behaviors [9-13]. However, most results are based on self-reported anthropometric measures and/or physical activity and sedentary behaviors.

Of dietary factors intake of sugar-sweetened beverages and breakfast consumption have been the most frequently studied associations [6,9,14-16]. Norwegian adolescents have been shown to have a higher intake of added sugar than recommended [17]. Watching TV is the most studied sedentary behavior, and some authors suggest that TV-viewing influences weight through the impact on energy intake rather than displacement of more energy demanding activities [18]. Computer use and video games do not seem to represent such a high risk compared to watching TV, as long as it does not replace physical activity [19]. More studies investigating the association between sedentary behaviors and weight status have been called for [20]. While some reviews state that there is no conclusive evidence for an association between body composition measures and children’s self-reported physical activity [12,21], some studies with accelerometer-assessed physical activity have shown that a higher BMI and percentage body fat are associated with less physical activity [22,23]. Yet, a recent meta-analysis of prospective studies reported no association between objectively measured physical activity and fat mass in children [24].

Both BMI and correlated behaviors tend to change over time and differ substantially by age group. The age of 10–11 years is called a “key transition age” in a preventive perspective [25], because adolescents are establishing behavioral patterns that may continue into adulthood and this has implications for long term health. In their systematic review of school-based interventions to prevent childhood obesity, Brown and Summerbell (2009) conclude that interventions seem to reach genders differently [26]. Further, a systematic review of cross-sectional studies investigating the association of socioeconomic status (SES) and childhood adiposity concludes that associations exist and are predominantly inverse [27]. Stratified by parental education level, Bjelland et al. (2011) found significant differences in anthropometric characteristics and prevalence of overweight in the HEIA study [28]. These results indicate a need to explore whether modifiable behavioral correlates of weight status differ by non-modifiable demographic variables such as gender and SES. The present study investigates adolescents at the start of puberty. To our knowledge few studies have been published that include both objectively measured weight, height, physical activity and sedentary time, which assess dietary information and screen time/sedentary behavior, and also include self-reported parental education on a large cohort of adolescents in the beginning of puberty.

The aim of this study was to investigate both modifiable correlates (dietary factors, sedentary behaviors, physical activity) and non-modifiable correlates (gender and parental education) of weight status among Norwegian 11-year-old adolescents.

## Methods

The HEalth in Adolescents Study (HEIA) study is based on a socio-ecological framework that aims to combine personal, social and physical environmental factors hypothesized to influence overweight and obesity in children, mediated by dietary and physical activity behaviors [29,30]. The design and procedure of the HEIA study are thoroughly described elsewhere [30], and thus only a brief description will follow.

### Study design and subjects

Eligible schools were those with more than 40 pupils in 6^th^ grade and located in the 3–4 largest towns/municipalities in the 7 counties surrounding the county of Oslo. Of 177 schools invited, 37 schools agreed to participate. All 6^th^ graders in these 37 schools (n = 2165) were invited to participate. Of these, 1580 adolescents accepted to participate and returned a parent signed informed consent form (73%).

The main data collections took place in September 2007 and were conducted by trained staff. On the day of the survey the participating adolescents took part in an examination of anthropometric measures, filled in an Internet-based questionnaire and a short paper questionnaire about pubertal status. In addition, physical activity was measured objectively by accelerometers. The physical activity data collection was performed separately from the main survey due to logistics, and took place from September until the beginning of December 2007.

A total of 1481 adolescents (94% of those 1580 returning a consent) provided anthropometric measures and completed the survey. Valid accelerometer data was provided by 1129 adolescents. Reasons for not being included in the accelerometer analysis were: not wearing the accelerometer (n = 247), failing to achieve at least three days of assessment (including at least one weekend day) (n = 40) and instrument malfunction (n = 23). The adolescents present at the day of the data collection with complete anthropometric measures and valid accelerometer data (n = 1103) are included in this paper. No differences in anthropometric data, weight status or parental education were observed between children with and without valid accelerometer data, but there were more boys in the group without accelerometer data and complete anthropometric data (p = 0.008) (data not shown).

Ethical approval and research clearance was obtained from the Regional Committees for Medical Research Ethics and the Norwegian Social Science Data Service.

### Anthropometric measurements

Height was measured to the nearest 0.1 cm, using a wall-mounted tape with the child standing upright against the wall without shoes. The adolescents’ weight was measured with light clothing (i.e. t-shirt and underwear) to the nearest 0.1 kg using a Tanita scale (Tanita TBF-300, Tanita Corporation of America, Illinois, USA). BMI was calculated as weight/(height x height) (kg/m^2^). The age and gender specific BMI cut-off values proposed by the International Obesity Task Force [31] were used to categorize the adolescents as non-overweight or overweight. The obese participants (1.8%) were included with the overweight in the analyses.

### Data from questionnaires

Intake of sugar-sweetened beverages was assessed by frequency (six categories; from never/seldom to every weekday) and amount (in glasses; from one glass to four glasses or more) for weekdays, and by amount for weekends (in glasses; eight categories; from never/seldom to seven glasses or more). Weekends were defined as Saturday and Sunday. In the questionnaire it was stated that 0.5 l of beverage was equal to three glasses, making one glass equal to 1.67 dl. Intake of snacks was assessed by four questions; how often do you eat chocolate/candy, salty snacks, cookies and buns/cakes/pastry with seven response categories from never/seldom to twice a day or more. All variables were recorded into frequency of intake per week by using the midpoints of the categories (making 1–2 times a week equal 1.5 times per week) and summed into a sum of snacks variable. Breakfast consumption was assessed by the question; how often do you eat breakfast, with nine response categories ranging from never to every day. Since 90% of the responses were “every day”, this variable was recorded to a dichotomous variable; eats breakfast every day or not. Two questions assessed hours of daily TV-watching (including DVD) and use of computer/electronic games on weekdays and weekends separately, each question with six response categories ranging from 0.5-5 and from 0–4 hours, respectively. The questions were mostly modified from existing questionnaires. Test-retest reliability of these self-reported behavioral outcomes showed moderate to high correlation (mostly r > 0.6) and is further described elsewhere [30].

The pubertal scale utilized in the study is based on the Pubertal Category Scores (PCS) [32]. PCS for boys included body hair growth, voice and facial hair. For girls, PCS included body hair growth, breast development and menarche. The adolescents were categorized into 5 groups, but due to low numbers in the last two categories, the final puberty score consisted of 3 categories (Pre, Early and Mid/Late/Post-pubertal). Test-retest of the puberty questionnaire showed a reasonable reproducibility (data shown elsewhere [30]).

As part of the informed consent, self-reported information about parental education was collected for both mothers and fathers. Parental education was categorized into three levels: 12 years or less of total education, between 13 and 16 years, and 16 years or more. The information about education from the parent with the longest education was used in the analyses, or else the one available.

### Sedentary time and physical activity measured by accelerometers

The children were instructed to wear accelerometers (ActiGraph GT1M/CSA model 7164, Fort Walton Beach, FL, USA) all waking hours for 5 consecutive days except when doing water activities (monitors are not waterproof, water activities were ignored). The output was sampled every 10 seconds for 2 weekdays and 2 weekend days. The registration was set to start the second day of wearing the monitors to avoid excessive activity likely to occur during the first day. Activity should be registered during a minimum of 3 days (including at least one weekend day) and at least for 8 hours (480 min) each day to be considered as acceptable use.

After collecting the accelerometer, the stored activity counts were downloaded to a computer and analyzed by a software program named “CSA-analyzer” (http://csa.svenssonsport.dk). In the analyses of accelerometer data only daytime activity (06:00–24:00 hours) was included. Sequences of 20 min or more of consecutive zero counts were interpreted to represent non-wear-time and were excluded from each individuals recording.

The average number of minutes that the participants wore the accelerometer and the number of activity counts per minute (cpm) were calculated. Mean cpm (mcpm) as a summary measure of total physical activity in children is commonly used and has been validated against the “gold standard measurement” doubly labeled water and found valid [33]. Since outcomes on mcpm measured by model 7164 and GT1M have been shown to differ [34], a free-living validation study of the monitors used in the HEIA study was conducted (Grydeland et al., unpublished observations). In accordance with results from Corder et al. (2007), model 7164 was shown to measure 11% higher total mcpm than GT1M and a correction factor of 0.9 was applied to the total mcpm from model 7164 to be comparable to the GT1M outcome.

Sedentary time was defined as activity at intensities less than 100 cpm and expressed as min/day of accelerometer activity measured, which equals the intensity of sitting or lying down (<1.5 MET) [35]. Activity recordings at intensities between 100–2000 cpm were defined as light activity, reflecting activities such as standing, walking slowly or easy play. Moderate to vigorous physical activity (MVPA) was defined as all activity at intensities above 2000 cpm. This threshold is approximately equivalent to a walking pace of 4 km/h in youth [36]. These cut off points have been used in previous studies [37,38]. Sedentary time, light activity and MVPA were expressed as min/day of accelerometer activity measured.

### Statistics

Clustering effects due to schools being the unit of recruitment were checked by Linear Mixed Model procedure (analyses available upon request). No clustering effect was found for the adolescent’s BMI, and only 2% of the unexplained variation was on group level. If there is no meaningful difference among groups when quantifying degree of clustering, data may be analyzed at individual level [39,40]. Additionally, pupils in Norwegian primary and secondary education are no longer organized by classes (but in larger and smaller groups varying by study subject) (Norwegian Education Act, 2003). Clustering effects of class are therefore not investigated. Based on these arguments we decided not to do multilevel analyses in this paper.

Anthropometric characteristics were presented as means and standard deviations (SD), unless otherwise stated. Continuous variables were tested for differences between genders and between weight categories with independent sample t-tests, and categorical variables were tested by chi-square tests. Paired samples t-test was used to test differences in continuous variables between weekdays and weekend days.

The associations of the modifiable correlates with weight category (non-overweight/overweight; above/below cut-off) were analyzed by univariate and multiple logistic regression by the Forced Entry Method, controlled for gender and pubertal status. Variables that were associated with weight status at a p < 0.10 in the univariate analyses were entered in the final model. The results are presented as crude and adjusted odds ratios (OR) with 95% confidence intervals (CI).

To test whether gender or parental education level moderated these associations, interactions between gender and education level and each factor were tested separately in the model. Significant interactions were further inspected.

The significance level was set to p < 0.05 for all analyses (interaction analyses p < 0.10). Data were analyzed using the PASW Statistics, version 18 (SPSS) (IBM Corp., New York, NY, USA).

## Results

Girls were slightly taller and heavier than boys, and had a higher puberty scale score than boys (Table 1). There were no differences in BMI or weight status between genders. No differences were found between genders for parental education. There were significant differences between genders in consumption of sugar-sweetened beverages and snacks, watching TV and playing electronic/computer games, where boys’ averages were higher than girls’ averages for all outcomes. There were also differences in these behaviors with regard to weekdays and weekend days. On average the accelerometers were worn for 784 ± 62 min/day (girls 779.3 (62.8) and boys 788.1 (60.8), p = 0.02). There were no gender differences in time spent sedentary and in light physical activity. Girls spent 7.8% of the monitored time in MVPA while boys spent 9.5% (p < 0.001). Boys showed more total physical activity than girls.

**Table 1:** **Descriptive characteristics for all, by gender and by weight status**^**a**^**in 11-year-old Norwegian adolescents**

**Characteristics**	**All**	**Girls**	**Boys**	***p***	**Non-overweight**	**Overweight/obese**	***p***
	**(n = 1103)**	**(n = 555)**	**(n = 548)**		**(n =962)**	**(n = 141)**	
Girls (% (n))	50 (555)				50 (482)	52 (73)	0.7
Age (years)	11.2 (0.3)	11.2 (0.3)	11.2 (0.3)	0.4	11.2 (0.3)	11.2 (0.3)	1.0
Weight (kg)	39.8 (8.0)	40.3 (8.1)	39.2 (7.9)	**0.03**	37.8 (5.9)	53.4 (7.4)	**<0.001**
Height (cm)	148.7 (7.0)	149.2 (7.3)	148.1 (6.6)	**0.01**	148.2 (6.9)	151.8 (6.9)	**<0.001**
BMI (kg/m^2^)	17.9 (2.7)	18.0 (2.7)	17.8 (2.7)	0.2	17.1 (1.7)	23.1 (2.1)	**<0.001**
Overweight/obesity ^**a**^ (% (n))	13 (141)	13 (73)	12 (68)	0.7			
Pubertal score (% (n))							
Pre-puberty	18.3 (202)	8.5 (46)	31.5 (156)		19.4 (176)	19.8 (26)	
Early-puberty	33.9 (374)	20.2 (109)	53.4 (265)		37.0 (335)	30.0 (39)	
Mid/late/post puberty	41.7 (460)	71.3 (385)	15.1 (75)	**<.001**	43.5 (395)	50.4 (66)	0.2
Parental education (% (n))							
≤12 years	29.1 (321)	30.2 (163)	29.6 (158)		28.1 (264)	42.5 (57)	
13-16 years	34.8 (384)	33.0 (178)	38.6 (206)		36.1 (339)	33.6 (45)	
>16 years	33.5 (369)	36.9 (199)	31.8 (170)	0.1	35.9 (337)	23.9 (32)	**<0.001**
SSB (dl/week)	10.2 (10.9)	8.7 (9.1)	11.7 (12.3)	**<.001**	10.1 (10.6)	10.9 (12.9)	0.5
SSB (dl/weekday)	1.1 (1.6)	1.0 (1.3)	1.4 (1.9)	**<.001**	1.1 (1.6)	1.3 (2.2)	0.3
SSB (dl/weekend day)	2.2 (1.9)	2.0 (1.7)	2.4 (2.1)	**.001**	2.2 (1.9)	2.2 (2.0)	0.7
Sum snacks (times/week) ^b^	4.5 (4.4)	4.1 (3.4)	4.9 (5.2)	**0.01**	4.5 (4.5)	4.2 (4.2)	0.5
Breakfast daily (% (n))	90 (996)	90 (499)	91 (497)	0.4	92 (876)	85 (120)	**0.01**
TV(hrs/day/week)	1.7 (1.0)	1.6 (0.9)	1.7 (1.0)	**0.005**	1.6 (0.9)	2.1 (1.2)	**<0.001**
TV(hrs/weekday)	1.4 (1.0)	1.4 (1.0)	1.5 (1.0)	**0.02**	1.4 (0.9)	1.9 (1.2)	**<0.001**
TV(hrs/weekend day)	2.2 (1.2)	2.1 (1.1)	2.3 (1.3)	**0.001**	2.1 (1.2)	2.6 (1.4)	**<0.001**
Computer game (hrs/day/wk)	1.2 (0.9)	1.0 (0.8)	1.4 (1.0)	**<.001**	1.1 (0.9)	1.4 (1.0)	**<0.001**
Computer game (hrs/weekday)	1.1 (0.9)	0.9 (0.8)	1.2 (1.0)	**<.001**	1.0 (0.9)	1.4 (1.1)	**0.001**
Computer game (hrs/weekend day)	1.5 (1.1)	1.2 (1.0)	1.8 (1.1)	**<.001**	1.4 (1.1)	1.7 (1.2)	**0.013**
Sedentary time (min/day)	490.0 (67.5)	493.8 (66.9)	486.1 (68.0)	0.06	488.4 (67.6)	500.5 (66.3)	**0.05**
Light activity (min/day)	225.8 (43.1)	224.9 (40.6)	226.9 (45.3)	0.5	225.6 (42.5)	225.2 (46.5)	0.9
MVPA (min/day)	67.5 (23.3)	60.6 (18.5)	74.5 (25.6)	**<.001**	68.4 (23.6)	61.5 (20.9)	**<0.001**
PA total (cpm)	512.3 (166.7)	474.9 (140.2)	550.1 (182.2)	**<.001**	517.9 (165.8)	473.7 (168.4)	**0.003**
PA weekday (cpm)	548.4 (178.9)	508.9 (164.2)	588.3 (184.3)	**<.001**	553.0 (180.6)	516.5 (163.5)	**0.02**
PA weekend (cpm)	463.2 (235.1)	428.8 (188.7)	498.0 (270.0)	**<.001**	471.3 (235.2)	407.7 (227.2)	**0.003**

Values are Mean (SD), except for overweight/obesity, puberty, parental education and breakfast % (n). The numbers (n) vary slightly for the different measures.

^a^ Age and gender specific cutoffs for overweight/obesity at age from 10.5 to 12.5 as defined by International Obesity Task Force [31].

^b^ Sum of intake of chocolate/candy, salty snacks, cookies, buns/cakes/pastry. *SSB* = sugar-sweetened-beverages, *MVPA* = moderate-to-vigorous physical activity, *PA* = physical activity, *P* = *t*-test/chi-square (between genders and weight status).

When the adolescents were categorized by weight status into groups of non-overweight and overweight/obese, there were no differences in gender or age between groups (Table 1). No differences were seen in puberty score by weight status, but a highly significant difference was seen for parental education (p < 0.001), with parents of overweight adolescents having less education.

Furthermore, Table 1 shows no differences between the two weight categories for dietary factors, except for breakfast consumption for which more non-overweight than overweight reported having breakfast daily.

Overweight adolescents spent more time watching TV and playing computer games than non-overweight adolescents. Both weight categories reported watching more TV and playing more computer games during weekend days than weekdays (p < 0.001). The overweight adolescents spent on average 37% and 21% more time in front of the TV than non-overweight during weekdays and weekend days, respectively (p < 0.001). Additionally, the overweight adolescents spent 32% and 19% more time playing computer games than non-overweight during weekdays and weekend days, respectively. Differences by weight status were seen for accelerometer data as well, with the non-overweight adolescents showing less sedentary time, more MVPA and total physical activity than the overweight.

Crude and adjusted logistic regressions for the factors potentially associated with weight status are presented in Table 2. Since variables for weekdays and weekend days on each of the studied behaviors were highly correlated (e.g. TV and computer use r = 0.7, p < 0.001), both variables could not be included in one regression analysis but were summed per week. Total physical activity (cpm) was highly correlated with both MVPA and sedentary time and was therefore left out of the analysis.

**Table 2:** Factors associated with being overweight/obese ^a^ in a group of 11-year-old Norwegian adolescents (n = 1103)

	**Crude**			**Adjusted**		
	**OR**	**95%CI**	***p***	**OR**	**95%CI**	***p***
Parental education ≤12 y	1			1		
13-16 y	0.62	0.40, 0.96	**0.03**	0.58	0.37, 0.92	**0.020**
>16 y	0.47	0.29, 0.75	**0.002**	0.54	0.33, 0.89	**0.015**
SSB (dl/week)	1.00	0.98, 1.02	0.93			
Sum snacks (times/week)	0.97	0.92, 1.03	0.30			
Breakfast daily (yes/no)	2.00	1.17, 3.40	**0.01**	1.78	1.01, 3.11	**0.045**
TV (hrs/day)	1.53	1.28, 1.82	**<0.001**	1.40	1.14, 1.72	**0.001**
Computer game (hrs/day)	1.43	1.18, 1.74	**<0.001**	1.18	0.94, 1.48	0.16
ST (min/day)	1.00	1.00, 1.01	0.07	1.00	0.996, 1.003	0.81
MVPA (min/day)	0.99	0.98, 0.995	**0.003**	0.99	0.974, 0.996	**0.010**

^a^ Age and gender specific cutoffs for overweight/obesity at age from 10.5 to 12.5 as defined by International Obesity Task Force [31]. Numbers are adjusted for gender and puberty.

Adolescents with parents in the highest education category (>16 years) had a 46% reduced odds of being overweight compared to adolescents with parents in the lowest education category (≤12 years) (p = 0.02). Adolescents of parents with medium education (13–16 years) had 42% lower odds of being overweight than adolescents of parents with the lowest education category (p = 0.02).

Consumption of sugar-sweetened beverages and snacks was not associated with weight status. Having daily breakfast was associated with weight status, both separately (OR 2.0) and adjusted for the other factors (OR 1.78).

Hours spent in front of the TV and playing electronic/computer games were both positively and significantly associated with being overweight in the univariate analyses. In the adjusted model only watching TV remained highly associated with being overweight, with a 40% increased risk of being overweight with every additional hour of watching TV per day.

Whereas MVPA was negatively and significantly associated with being overweight in the adjusted model, objectively measured sedentary time was not associated with being overweight.

Investigating whether gender moderated these associations, interactions between gender and each of the factors in the multiple model were tested. The only significant interaction was between gender and watching TV; OR 1.75 (CI 1.50, 2.65) p = 0.009. Sub-group analyses by gender revealed that the association between weight status and watching TV was highly significant for boys (OR 2.1 (95% CI 1.58, 2.73) p < 0.001) but not for girls (OR 1.2 (95% CI 0.90, 1.53) p = 0.23).

Investigating whether parental education moderated the investigated associations, interactions between parental education and each of the factors in the multiple model were tested. No significant interactions were found.

## Discussion

The main findings in this study were that level of parental education, daily breakfast consumption and MVPA were inversely associated with weight status, and time spent watching TV was positively associated with weight status in a sample of Norwegian 11-year-olds. The association between watching TV and weight status turned out to be significant for boys only.

The prevalence of overweight and obese adolescents in the present study does not differ substantially from other recent studies of Norwegian adolescents [6,9,14,41].

The lack of association between sugar-sweetened beverages and weight status may be surprising given that WHO has categorized it as a probable contributor [4]. However, the intake of sugar-sweetened beverages was low in both groups, which has also been observed in another recent Norwegian study [42]. Yet, underreporting by the overweight cannot be ruled out, especially due to the high awareness about the sugar-sweetened beverages and health.

The association between weight status and consumption of unhealthy snacks was not significant. As a matter of fact, overweight adolescents tended to eat less snacks than non-overweight. This finding might be explained by underreporting or changed dietary patterns by overweight adolescents as a consequence of dieting. However, prospective studies of snack food consumption have consistently failed to show a link between snack food intake and excess weight [43]. Our results add to these findings.

An association between breakfast consumption and weight status has been identified in other cross-sectional studies [6,9,14]. We found a significant negative association in the univariate analyses, and also when adjusting for other factors. Affenito et al. (2005) found a negative association between breakfast consumption and BMI after adjusting for demographic characteristics in the NHLBI Growth and Health study, but the association did not persist after multivariate control for physical activity and energy intake [44]. The authors of the study interpreted this observation to suggest that breakfast consumption is a marker for other healthy behaviors. A large US longitudinal study reported that normal-weight children who never ate breakfast gained weight relative to peers who ate breakfast nearly every day [45]. Must et al. (2009) points out that breakfast consumption as well as consumption of sugar-sweetened beverages and TV-viewing seem to be operating indirectly, as proxies for other dietary or activity behaviors, and warrants further studies of these relationships [43].

Regarding time watching TV and playing computer games, the differences between non-overweight and overweight adolescents were quite high, especially during weekdays. Watching TV was strongly associated with being overweight in our study, with 40% increased odds of being overweight with every additional hour of watching TV per day in the total sample. However, gender moderated this association and the subgroup analyses revealed a significant association for boys only. A review from prospective studies on modifiable risk factors in relation to changes in BMI and fatness concluded that sedentary behaviors effect on weight status seems to differ by gender, with many studies but not all showing greater positive associations among girls [43]. Possible gender differences are important to be aware of when designing intervention efforts targeting overweight/obesity prevention. Watching TV is the most studied sedentary behavior, and some studies suggest that TV-viewing operates through the impact on energy intake rather than displacement of more energy demanding activities. Cross-sectional data from the Danish part of European Youth Heart Study showed inverse associations between watching TV and both healthy food preferences and healthy food habits in school aged children [46]. In a laboratory-based study including 9 to 14-year-old boys, watching TV during a meal seemed to delay normal mealtime satiation and reduce satiety signals from recently consumed food, increasing energy intake [18]. It has also been suggested that exposure to commercials advertising energy-dense food while watching TV can work as an indirect mechanism and increase the energy intake even more [13]. However, in Norway there are legislations restricting such TV commercials aimed at children and this issue should only be of limited importance.

We found a strong association between use of computer games and weight status in the univariate analysis, but this association did not remain when adjusting for the other factors, indicating a confounding effect of other variables on this relationship. Tremblay et al. (2011) found a dose–response relationship between increased sedentary behavior and unfavorable health outcomes in a large systematic review of sedentary behavior on health indicators based on 232 studies of school-aged children and youth [47]. The results are based on data from almost 1 million young people. However, these are self-reported sedentary behaviors measured as a mixture of hours/minutes/times per week of watching TV/playing computer games/screen time or self-reported sedentary time. A Canadian study using an objective measure of sedentary behavior (Actiwatch) found that fat mass and percentage body-fat were positively correlated with time spent sedentary for girls but not boys [48]. We found no significant association with time spent sedentary measured by accelerometer and weight status. The lack of consistency observed in the studies of sedentary behavior and sedentary time may reflect the range of variable definitions, measurement challenges, and also the changing nature of electronic media.

For total physical activity (mcpm) we found a significant difference between genders and between non-overweight and overweight adolescents. We found a significant inverse association between MVPA and being overweight, but no moderating effect of gender on this association. Ekelund et al. (2012) combined data from multiple cohorts of accelerometer assessed physical activity of 20 871 children and adolescents (age 4–18 years) and found that both total physical activity and time in MVPA were significantly and negatively associated with waist circumference [49]. However, in a systematic review of prospective studies of objectively measured physical activity and obesity prevention in children, adolescents and adults, the authors conclude that physical activity might not be a key determinant of excessive gain in adiposity [50]. Currently, the literature is inconsistent in the relationship between physical activity and adiposity.

Earlier findings from the HEIA study showed differences in anthropometric characteristics and prevalence of overweight when stratified by parental education level [28]. The current results show that these differences by parental education level remain when adjusting for behavioral factors. Level of parental education was inversely associated with weight status in the adjusted model, and this association was not moderated by gender. These results confirm previous studies suggesting a social gradient in the problem of overweight/obesity among Norwegian adolescents [6,27,51]. While earlier international research (from 1941–1989) found inconsistent relationships between SES and childhood adiposity [52], more recent research indicates a shift in trends where most studies show inverse relationships [27]. The ENERGY Project, a recent school-based survey among 10–12 year olds conducted in seven European countries (n = 7234), found more favorable indicators of weight status in children of higher educated parents than in children of lower educated parents [15]. Our results support the evidence that adolescents of parents with low education have a higher risk of being overweight than adolescents of parents with higher education independent of behavioral correlates and gender.

### Limitations and strengths

There are several limitations that should be considered when interpreting the findings from this study. It is impossible to infer causal relationships and to determine its direction from cross-sectional data. We cannot be certain that other unmeasured confounders could not have impacted our findings. Although accelerometers are considered a preferred tool when assessing physical activity and sedentary time in large surveys, it has weaknesses when it comes to measuring water-activities, cycling, skiing/skating, carrying loads, inclines and upper-body movements. We made no attempts to correct for these weaknesses, as to our knowledge there are no valid techniques to do so for a large scale survey. This may represent a possible underreporting of total physical activity. Furthermore, there was a rather large proportion of invited schools that declined to take part in the study. Recruiting schools in Norway to extra-curricular projects has become a challenging undertaking the last decade, as the curricular demands the last years have increased substantially. In addition, weighing of children is a controversial issue in Norway and has been debated in the national media repeatedly. However, attrition analyses showed no differences between the participating schools (n = 37) and schools which declined to participate (n = 140) in terms of number of students in 6^th^ grade and overall size (data not shown) [53]. Also, the sample is collected from seven counties surrounding the county of Oslo, and this may limit the possibility to generalize the results for 11-year-olds outside this area. However, comparing the HEIA study sample to nationally representative figures for 9 and 15-year-olds, the measures from the participants in the HEIA study lie adequately between the measures of the 9 and 15-year-olds when it comes to objectively measured height, weight and total physical activity [54].

One of the major strengths of the study is the relatively large sample of adolescents at a very narrow age range. The age of 10–11 years is an important age group for addressing efforts to promote healthy behaviors [25]. The fact that many adolescents at this age have entered puberty makes this specific age group considered as hard to study and therefore less studied than pre- and post-pubertal adolescents. We adjusted for pubertal development and taking this into account is one of the strengths in this study. Other strengths of the study are that we measured height, weight, physical activity and sedentary time objectively. All adolescents in the present study had valid accelerometer measures of physical activity and sedentary time. An additional strength is that we were able to collect data on parental education from nearly all the parents giving their consent for their child to participate in the study. Finally, investigating both behaviors related to energy intake and expenditure in the same study concerning weight status can be considered as a strength, as the intrinsic interplay among dietary behaviors, physical activity and sedentary time still needs further understanding.

## Conclusions

The present study shows that parental education, breakfast consumption, MVPA and TV-viewing were associated with weight status in a sample of Norwegian 11-year-olds. The social gradient in overweight remains a challenge for future interventions to target. While parental education cannot be regarded as a modifiable correlate of adolescents’ weight status, breakfast consumption, MVPA and watching TV can, and should be properly addressed in future interventions targeting overweight in this age group.

## Abbreviations

HEIA: HEalth in Adolescents; BMI: Body mass index; IOTF: International Obesity Task Force; SES: Socioeconomic status; PCS: Pubertal Category Scores; cpm: Counts per minute; MVPA: Moderate to vigorous physical activity.

## Competing interests

The authors declare that they have no competing interests.

## Authors’ contributions

MG worked on the statistical analyses, wrote the first draft of the manuscript and made the greatest contribution to the paper. MG, IHB, MB, NL, LFA, YO, KIK and SAA participated in designing the study and project planning. NL was the project coordinator and participated in all parts of the work. KIK initiated the study. All authors provided critical revision of the paper, and read and approved the final manuscript.

## Pre-publication history

The pre-publication history for this paper can be accessed here:

http://www.biomedcentral.com/1471-2458/12/1053/prepub
